# Replacement of Soya DOC by rice distiller's dried grains with solubles supplemented with protease enzyme and its effect on performance, nutrient metabolizability, carcass traits and gut health of broiler chicken

**DOI:** 10.5713/ab.23.0533

**Published:** 2025-08-12

**Authors:** Roshan Lavakumar Werulakar, Atul Parashuram Dhok, Sudhir Bhimrao Kawitkar, Nitin Vasantrao Kurkure, Pratiksh Kaluram Bacche, Mahesh Ravindra Jawale, Shital Vitthalrao Chopde, G. Roupesh, Darshana B Bhaisare, Madhuri S Hedaoo, Shilpa V Shinde, Shweta Ramesh Lende

**Affiliations:** 1 Department of Animal Nutrition, Nagpur Veterinary College, Nagpur, India; 2 Maharashtra Animal and Fishery Sciences University, Nagpur, India; 3 Department of Poultry Science, Nagpur Veterinary College, Nagpur, India; 4 Department of Veterinary Pathology, Nagpur Veterinary College, Nagpur, India; 5 Department of Veterinary Public Health, Nagpur Veterinary College, Nagpur, India

**Keywords:** Broiler Chicken, Gut Health, Protease, Rice Distiller's Dried Grains with Solubles

## Abstract

**Objective:**

The study was conducted to utilise rice distiller's dried grains with solubles (DDGS) as a protein source in broiler chicken supplemented with protease enzyme and also to assess its effect on growth performance, nutrient metabolizability, carcass traits, gut health and economics of broiler production.

**Methods:**

The study was conducted on three hundred day-old straight-run commercial broiler chicks which were divided into five groups of four replications each and fed as CG control receiving a standard basal diet;15- rice-based distillers' dried grains with solubles (RDDGS) with 15 percent rice DDGS;15-(rice distillers dried grains with solubles supplemented with enzyme)RDDGSE with 15 percent rice DDGS supplemented with protease enzyme @ 300g/ton of feed;20-RDDGS with 20 percent rice DDGS and 20-RDDGSE with 20 percent rice DDGS supplemented with protease enzyme @ 300 g/ton of feed.

**Results:**

The final body weights were higher (p<0.01) and FCR was better (p<0.05) for CG and comparable with 15-RDDGS and 15-RDDGSE. The CP metabolizability, was higher (p<0.05) for CG than 15 and 20 percent rice DDGS groups; however, the CP metabolizability for protease supplemented 15 and 20 percent rice DDGS groups was comparable with control diet. The total viable count and *Escherichia coli* count revealed non-significant differences. There was no negative impact on histological changes in intestine due to the inclusion of rice DDGS. There was no negative impact on histological changes such as crypt depth, villus width and villus height in intestine due to interaction between DDGS and enzyme. The carcass traits revealed non-significant differences for dressing percentage, overall giblet weight and abdominal fat.

**Conclusion:**

It was concluded that rice DDGS can be incorporated up to 20 levels in broiler diet without any adverse effect; however, inclusion of 15 percent rice DDGS supplemented with protease enzyme proved to be more economical in broilers.

## INTRODUCTION

The poultry diet contains a substantial amount of cereals and edible oil seed meals, which directly compete with human consumption. Because there is nearly no more room for agriculture, the availability of feed resources could be one of the biggest obstacles to increasing poultry production in the future. Scientists have been forced to look for alternate sources of feed components due to the rising expense of conventional animal feed ingredients in the majority of developing nations. In emerging nations, there is a greater need for alternative ingredients, which has a significant impact on the need for feed and raw materials. Important components of poultry feed are corn and soybean meal. The cost of producing feed has increased as a result of the ever-increasing demand and decreasing supply of raw feed ingredients. There are two ways to lower the cost of production: one is to reduce feed costs, which appears challenging given the rising costs of feed ingredients, and the other is to investigate new feed ingredients that are less expensive, locally accessible, and comparable in terms of their nutrient contents to currently used feed ingredients.

Today, modern rice byproducts that can be used by rice processing businesses are accessible in significant numbers and at lower prices, such as rice-based distillers’ dried grains with solubles (rDDGS). It is a fairly recent feed ingredient with a brownish colour and a coarse, powdery texture. The distiller’s dried grains with solubles (DDGS) are by-products of both the bio-ethanol and spirit industries. They are produced by drying mash over an extended period of time after multiple stages of concentration. It has a significant amount of additional protein (47%), and it has about 3,500 kcal/kg of metabolizable energy. Protein, exogenous amino acids, B-group vitamins, biotin, and mineral components, including phosphorus, are all abundant in the dried grains of cereal distillers [1,2]. Rice is processed by being heated at 131°C and 2.6 kg/m^2^ pressure, then having yeast added for fermentation [3]. With the exception of the majority of the starch, which has been consumed during the fermentation process, it contains all the nutrients from grain in a concentrated form. Following the fermentation stage, the subsequent step involves the distillation process. In the co-product recovery phase, subsequent to distillation, the non-volatile components, referred to as whole stillage, undergo centrifugation. This separation yields a liquid fraction, termed thin stillage, and a solid fraction known as distiller’s wet grains (DWG). A notable proportion, typically exceeding 15%, of the thin stillage is recycled as back set. This recycled thin stillage serves the purpose of being processing water for creating slurry with the ground grain. The remaining thin stillage undergoes concentration through evaporation, resulting in the formation of condensed distiller solubles. These condensed distiller solubles are then amalgamated with DWG to give rise to DWG with solubles. Following this, the amalgamated mixture is subjected to drying to produce the final product, DDGS. Even though there are few publications on the use of protease in broiler diets containing DDGS, in general, protease has been employed mostly in combination with other enzymes to increase the broiler chicken’s performance [4]. To improve the availability of the nutrients in the feed protease is supposed to elevate protein availability as endogenous enzymes are not sufficient to take care, hence the present study was undertaken to utilise rice DDGS with protease enzyme supplementation in broiler chickens.

## MATERIALS AND METHODS

The research work was approved by the Institutional and University Board of Studies vide Resolution No. 3/29. The experiment was conducted for six weeks on 300 day-old straight-run commercial broiler chicks (Vencobb 430) at Poultry Research and Training Centre, Nagpur Veterinary College, Nagpur during 18^th^ October to 29^th^ November 2022. The broiler chicks were divided into five groups of four replications each and fed as CG control receiving standard basal diet as per [5]; 15-RDDGS with 15 percent rice DDGS; 15-RDDGSE having 15 percent rice DDGS supplemented with protease enzyme; 20-RDDGS with 20 percent rice DDGS and 20-(rice distillers dried grains with solubles supplemented with enzyme)RDDGSE having 20 percent rice DDGS supplemented with protease enzyme. The enzyme protease was procured from Kemin Industries South Asia, Chennai marketed as KEMZYME and supplemented to chicks through feed @300 mg/kg feed. The protease was mixed uniformly with feed. Each treatment group was divided into four replicates of 15 birds each. All the birds were reared on deep litter system with four inches layer of paddy husk on the floor under identical management conditions and were fed in three phases as pre-starter (0–1 week), starter (1–3 weeks) and finisher (4–6 weeks) as per [5] specification (Table 1).

The experimental chicks were vaccinated against New Castle Disease through intraocular route on 7^th^ day with B1 strain, Infectious Bursal Disease (IBD) on 14^th^ day of age by intraocular route and booster vaccination of IBD invasive intermediate strain (B2K) was carried out on 21^st^ day and vaccination of New Castle Disease was done with *Lasota* strain on 28^th^ day.

Broiler chicks were weighed individually every week up to six weeks of age to determine the weekly body weight gain. The record of daily feed consumption was maintained to calculate weekly and total feed consumption. The feed conversion ratio was calculated from the ratio of amount of feed consumption and weight gain during that particular week or the cumulative FCR was calculated from the ratio of total feed consumed and weight gain until that respective week.


(1) $$\text{FCR}=\frac{\text{Amount of feed consumed (g) during the period}}{\text{Gain in body weight (g) during the period}}$$

A metabolic trial with 3 days collection period was conducted at the end of 6^th^ week by employing two birds from each replicate and 8 birds in a group to assess nutrient metabolizability. The pooled samples of feed offered and the faeces droppings collected during the metabolic trial were stored in the laboratory analysis and the representative samples of feed and dropping were then analyzed for proximate composition as per [6].

The moisture content of the sample was estimated by heating in an oven to constant weight at 100°C–105°C under atmospheric pressure. The constant weight of a sample after complete removal of moisture was dry matter.


(2) $$\text{Moisture }\%=\frac{\text{Loss in weight}}{\text{Weight of sample}}\times 100$$

The crude protein content was estimated as per Kjeldahl’s method by digesting 1 g dried sample with 30 mL concentrate H_2_SO_4_ adding 5 g of digestion mixture made of sodium sulphate and copper sulphate in 9:1 proportion. The digestion was carried for 2 to 3 h until it becomes clear. The digested content was then transferred to 100 mL volumetric flask with several washings with distilled water and 100 mL volume was made. Ten mL aliquot was then transferred to distillation assembly also adding 15 mL of 40% NaOH solution to the distillation unit. Released ammonia vapours were trapped in 15 mL of Tashiro’s indicator, which was prepared as 2% boric acid in 1,000 mL, adding 200 mL absolute alcohol, 12 mL methyl red (0.1%) and 6 mL bromocresol green (0.1%) solution. The ammonia boric acid complex (ammonium borate) was titrated with standard N/10 H_2_SO_4_ solution. The crude protein content was determined as per following formula assuming 1 mL of N/10 H_2_SO_4_ = 0.0014 g nitrogen.


(3) $$\text{Crudeprotein }\%=\frac{\text{Vol. of N}/10\;{\text{H}}_{2}{\text{SO}}_{4}\times 0.0014\times 6.25\times \text{Aliquot prepared}}{\text{Aliquot taken for distillation}\times \text{Weight of sample}}$$

The ether extract was determined by extracting 3 g dried sample with petroleum ether (60°C to 80°C) in Soxhlet’s assembly for about 7 h. After extraction, the extraction flask was placed in a hot-air oven at 100°C for 1 h, cooled it in a desiccator and weighed. The material recovered in the receiver flask is the weight of fat extracted by the petroleum ether and the extract was calculated as per following formula.


(4) $$\text{Etherextract }\%=\frac{\text{Weight of fat}}{\text{Weight of sample}}\times 100$$

Crude fiber was determined by refluxing 2 g moisture and fat free sample in spoutless beaker by adding 200 mL of 1.25% sulphuric acid for 30 min, timed from onset of boiling and then with 200 ml of 1.25% NaOH solution. The contents were then filtered through the muslin cloth. The residue taken in a crucible was placed in the hot-air oven overnight at 80°C to 110°C until the constant weight was achieved. The residues were then ignited in a muffle furnace at 600°C for 2 to 3 h, cooled and weighed again. The loss of weight due to ignition was the weight of crude fiber. The crude fiber was calculated using following formula:


(5) $$\text{Crude fiber}\%=\frac{\text{Weight of crude fiber}}{\text{Weight of sample}}\times 100$$

Nitrogen-free extract was obtained as NFE = 100−(CF %+CP %+EE %+Ash %).

For carcass study at the end of the 6^th^ week one male and one female bird from each replicate of group were randomly selected, starved for 12 h before the slaughter, however drinking water was provided *ad libitum*. The birds weighed accurately and then scarified by severing the jugular vein and carotid artery on one side of the neck, allowed to bleed for 1 to 2 min. The dressed weight of each group was obtained separately after complete bleeding and removal of feathers, viscera, head and legs by keeping the skin intact with the carcass. The dressing percentage, giblet weight (liver, heart, and gizzard), and abdominal fat were expressed as % of live weight.

For determination of total viable count (TVC) and coliform count, large intestine was opened immediately after sacrificing and 1 g of caecal content was collected in sterile glass vial and diluted in 9 mL normal saline and then 2 serial dilutions were made for inoculation. Then, 10 μL content from the last test tube was poured on the EMB agar plates for *Escherichia coli* count and on the nutrient agar for TVC, and then kept for incubation for 24 h at 37°C. The average number of colonies in a particular dilution was multiplied by the dilution factor to obtain the TVC. The results of the total bacterial count were expressed as the number of organism of colony forming units per gram (CFU/g) of caecal samples. The colony-forming unit in respect of *E. coli* was carried out for caecal contents by serial dilution method as per standard protocols of Ragione et al [7]. After serial dilution, the spread-plating method was employed to distribute the inoculum on the EMB agar plate. Further, the EMB plates were incubated at 37°C overnight. The colonies resembling *E. coli* were counted and values were converted to CFU per gram of weight.

The histo-morphology of the small intestine was determined in the intestinal segments (duodenum, jejunum, ileum) obtained during the slaughter of the animals. The villus height (VH), crypt depth (CD), and the thickness of the muscularis mucosa were recorded. The VH was determined from the tip of villi and top of lamina propria and the crypt depth was measured from the base to the region of transition between the crypt and the villus. For this purpose, the tissue samples of the intestine were fixed in 10% buffered formalin immediately on removal. The segments of the small intestine were washed with the normal saline solution to remove blood and tissue debris. The intestinal contents were emptied and the segments were washed again. The section of the small intestine was cut into small pieces not exceeding 2 mm in length and immersed into the alcoholic Bouin’s fluid (the fluid contained 150 mL of 80% ethyl alcohol, 1 g picric acid and 60 mL formaldehyde; to this stock solution 50 mL glacial acetic acid was added). After a period of 16 h the fixative was poured off and the tissue was washed thoroughly with 70% alcohol and dehydrated in ascending grades (80% and 90%) of absolute alcohol. The tissue segments were then immersed in a solution containing alcohol and cedar wood oil for 48 h and transferred to a solution containing xylene and paraffin wax at a ratio of 1:1 and kept in an incubator at 56°C to 58°C for 2 h. A quantity of molten paraffin was poured in small blocks into which the tissues were transferred. The paraffin was allowed to harden and cut into suitable blocks from which tissue sections were cut at 5–10 μm with the help of microtome. The sections were placed on glass slides and kept in a hot plate to get the section adhered to the slide. The slides were then stained with Haematoxylin and Eosin (H&E) for histopathological observation [8] and mounted in DPX. For each specimen at least 10 fields were considered to be measurable unit during calculation.

The economics of broiler production was worked out by considering the total cost of production which included the cost of feed, chicks, protease, vaccines, medicine and other expenditure. The profit per kg live body weight was calculated after selling of the birds on live body weight basis in the local market. The data on various parameters viz. weekly body weight gain, feed consumption and feed conversion ratio, nutrient metabolizability, carcass traits, TVC count, *E. coli* count, and intestinal morphology were analyzed as per IBM SPSS Statistics SPSS Base 8.0 version for Windows 2018 [9] and means were compared with Duncan’s multiple range test.

## RESULTS AND DISCUSSION

### Nutrient composition

The rice DDGS used in this experiment contains CP (50. 8%), CF (3.7%), EE (4. 9%), lysine (0.64%), methionine (1.19%) and ME (3,400 kcal/kg). The similar composition for rice DDGS was reported by Dinani et al [10], but the composition is higher than reported by Martinez Amezcua and Parsons [11] as CP (44.7%) and ADF (12.7%). Also researchers [12–15] reported lower protein levels for rice DDGS; however, Ranjan et al [16] reported higher CP content for rice DDGS. The drying process can have crucial influence not only on variability of nutrients in different samples but also palatability. The variations in chemical composition of rice DDGS may be attributed to extraction process, drying process and solubles content.

Several factors affect the nutritional and physical characteristics of DDGS causing variability. This includes the variability of nutrient levels in the sources, proportion of distillers soluble added to DDG before drying [11], efficiency of converting starch to ethanol and temperature and duration of drying [17]. Olentine [17] reported that lysine content in 5 sources ranged from 0.48% to 0.76% with the lowest lysine content in the darkest DDGS source. Since the DDGS sample used in the present study was dark in colour, it is pertinent to mention that Batal and Dale [18] observed considerable differences in the true amino acid digestibility among samples, and reported that more yellow and lighter samples were, in general, characterized by higher total and digestible amino acid levels, especially lysine. They suggested that the reason for the significantly lower total and digestible lysine content in the darker DDGS samples could be due to the destruction of a significant amount of lysine during excessive heating (during the Maillard reaction between reducing carbohydrate as glucose and the epsilon amino group of lysine). They also suggested that colour analysis might be a quick and reliable method of estimating the amino acid, particularly lysine, digestibility of DDGS for poultry. The proximate composition of various feeds was determined and presented in Table 2. The feeds were iso-nitrogenous and iso-caloric as per specifications of [5].

### Body weight gain

The body weight gain of broilers (Table 3) between the different groups varied significantly. During all the days, the total weight gain in CG group was higher (p<0.01) than other groups; however, during starter and finisher total body weight gain for control group was comparable with 15-RDDGSE. The body weight gain for 20-RDDGS group during all the weeks found to be lowest amongst all. Considerably lower body weight was found when broilers were fed rice DDGS [16,19]. However, researchers [19,20] previously found no statistically significant change in cumulative body weight gain when broilers were fed rice DDGS at 5%, 10%, 15% and 20% level. The comparable and positive effect of r-DDGS observed on body weight at the lower level in the present study could be attributed to the fermentation solubles and yeast residues present in DDGS, which were known to favourably influence the performance of chicks when fed in the diet [21]. Absence of such beneficial effect at the higher level of r-DDGS may be because these performance-enhancing attributes present in DDGS (like mannan in the yeast biomass) operate to their optimum at a particular concentration in the diet. However, body weight gain tended to be increased due to protease enzyme supplementation, whereas the differences were comparable, as the protease supplementation could degrade complex proteins in the diet into usable amino acids and peptides thereby resulting in improved protein digestibility and growth performance.

The body weights due to protease supplementation both in 15% and 20% RDDGS groups increased comparatively over the plain RDDGS; however, the differences were statistically non significant. The final body weights were higher (p<0.01) for control group and comparable with 15% RDDGS group with and without protease supplementation. It was also observed the supplementation of enzyme protease seems to have positive effects on body weights in each group either with 15% or 20% inclusion of RDDGS.

The present findings on body weights are consistent with Dingore [15] who observed lower (p<0.05) body weights with higher levels of rice DDGS in broiler chickens. Lower body weights were reported due to inclusion of 15% rice DDGS in broilers [10]; however, for 7.5, 10 and 12.5 percent inclusion of rice DDGS the body weights were comparable with control diet. On the contrary, Singh et al [12] found improved body weights in broilers fed rice DDGS at 10 and 15 percent level of inclusion, as compared to control diet. Improved body weights were found due to rice DDGS in layers at 5, 7.5 and 10 percent level with protease supplementation [22]. However the body weights were not affected by the inclusion of 10% rice DDGS in broiler [20]. It is indicated that RDDGS supplementation in broiler diets with or without enzymes significantly improved the body weight gain of birds compared to control and attributed to high protein in RDDGS and also exogenous enzyme supplementation which could enhance the digestibility of nutrients leading to better performance [23].

The differences in body weights by different workers may be attributed to various grain DDGS sources, nutritional values of DDGS sources and the different levels of DDGS used in the study as supported by Dang et al [24]. Also Thein et al [19] opined that the different findings in growth performance of his experiment and other research might be due to many reasons as the source and processing method of rice DDGS, quality of rice DDGS and the differences in age feeding stage (starter or grower stage) of broiler chickens. The rice DDGS contained generally 65% distillers grain and 35% distillers solubles on dry matter basis. The AAFCO [25] reported that dry milling process leads to the Maillard reaction and further reduced the lysine availability as compared to parent grains used for ethanol production. Therefore, the different drying methods might change the quality of rice DDGS that is used for feed ingredients in poultry diets. Mir et al [26] pointed that lower amount of available lysine due to drying processing method during rice DDGS production can hamper the growth and efficiency. Lumpkins [27] also explained the growth depressing effect of DDGS in the more sensitivity low density diet. This might be the reason for comparatively lower body weights at higher level of rice DDGS in present study, although extra lysine and methionine were supplemented in the diet prepared with rice DDGS.

The comparable and positive effect of r-DDGS observed on body weight at the lower level in the present study could be attributed to the fermentation solubles and yeast residues present in DDGS, which were known to favourably influence the performance of chicks when fed in the diet [21]. Absence of such beneficial effect at the higher level of r-DDGS may be because these performance-enhancing attributes present in DDGS (like mannan in the yeast biomass) operate to their optimum at a particular concentration in the diet.

The body weight gain tends to be increased due to protease enzyme supplementation; however, the differences were comparable, as the protease supplementation could degrade complex proteins in the diet into usable amino acids and peptides thereby resulting in improved protein digestibility and growth performance. In addition, protease is capable of degrading grain storage proteins and liberating higher levels of available amino acids and energy and eliminates the effects of anti-nutritional factors [28]. Further, if the basal diet had a high nutrition level which had satisfied the nutrition requirement of animals, the supplementation of protease would not show positive effects on growth, while the basal diet had a low nutrition level or consisted of ingredients with relatively low quality, the improvement of growth by dietary protease could be observed. Hence the supplementation of protease could not elevate body weight gain significantly in the present study since diets were iso-caloric and iso-nitrogenous.

The by-product of processing rice known as rice DDGS is frequently utilised as a component of broiler chicken feed. Although rice DDGS is a good source of protein and energy, it may also contain anti-nutritional components such fiber, phytic acid, and tannins that might hinder the digestion and absorption of nutrients by birds. According to studies, broilers can have reduced body weight gain, feed intake, and feed efficiency when fed a diet high in rice DDGS. These anti-nutritional components, which can bind to nutrients in the meal and render them inaccessible to the bird’s digestive system, are most likely to blame for this. In order to support appropriate growth and development, the bird might not be able to extract enough nutrients from the meal, which would cause a decrease in body weight.

### Feed consumption

The total feed consumption varied between the groups except for pre starter. The total feed consumption for control group was higher (p<0.01) amongst all the groups. The protease supplementation could not elevate overall feed consumption on inclusion of 15% RDDGS. The lower feed consumption due to inclusion of rice DDGS is supported by Dinani et al [10] who reported that at 15% level of rice DDGS the feed intake reduced significantly. According to [29], there was no significant difference in feed consumption in the birds receiving diets containing DDGS up to 10% and 12%. Similar reduction in feed intake was observed by Hassan and Al-Aqil [29] who reported that the use of 20% DDGS in the diets significantly depressed the feed intake of birds. The findings of the present study are in agreement with the findings of Zhang et al [30] who noted similar feed consumption in broilers. They stated that broiler performance was severely impacted by feeding 10% DDGS and diets containing 20% DDGS reduced the growth phase and average daily feed consumption. Physical form of the diet may also influence the acceptable level of DDGS in broiler diets. Birds attempt to consume feed to meet their metabolic energy requirement. However diets with higher levels of DDGS had a lower bulk density, which may induce the feeling of fullness before meeting their energy needs [31]. Furthermore rice DDGS is unpleasant because it smells strongly of residual ethanol and has a bitter taste. As a result, there may be a decrease in the amount of feed consumed. Palatability concerns, high fiber content, unbalanced nutrient levels, and inadequate digestibility may all contribute to broiler’s low feed consumption when fed a diet high in rice DDGS. Also DDGS with a fine texture or small particle size can cause broilers to consume less feed. This is due to the ease with which the small DDGS particles can consolidate in the feed, resulting in a dense texture that is challenging for birds to ingest. Furthermore, the small particle size may result in dustiness and clumping, which may further lessen the feed’s palatability and decrease feed intake. Moreover supplementation of protease has increased cumulative feed consumption significantly for 20% rice DDGS. Proteases can optimize feed protein implementation in poultry and proteases employed as feed additives could serve to supplement the effects of endogenous pepsin and pancreatic enzymes via the augmentation of hydrolysis and solubilization of protein. Also the increasing feed intake due to the concentrations of protease in the diet, attributed to the increased availability of amino acids that promote consumption.

### Feed conversion ratio

The FCR was significantly better for CG group and comparable with group 15-RDDGSE. The FCR for 15-RDDGS and 15-RDDGSE was comparable and for 20-RDDGS and 20-RDDGSE. Although the FCR of CG was better but for other groups also it was as per standard FCR as that of commercial broilers, indicated that the rice DDGS groups performed better with respect to FCR without any adverse effect and can be a better alternative protein source to soya DOC. The trend observed in the present study with regards to cumulative FCR is consistent with Dinani et al [10] who reported lower FCR when rice DDGS was incorporated in the diet at a level of 15%, however numerically the FCR values reported by them on 7.5% rice DDGS (1.79), 10% rice DDGS (1.81), 12.5% rice DDGS (1.80) and 15% rice DDGS (1.84) are poorer than reported in the present study. The FCR of 1.55 reported in the present study on inclusion of 20% RDDGS indicates better performance of broilers, feed and quality of rice DDGS. Similarly, Dingore [15] also reported lower FCR in broilers when fed rice DDGS, however values are poorer than reported in the present study, as 10% rice DDGS (1.95) and 15% rice DDGS (2.00). Even Khose et al [13] also observed poorer FCR numerically on 5, 10 and 15% inclusion of rice DDGS in broilers. They also reported numerically better FCR on supplementation of multi-enzymes on rice DDGS based diet; however, the values reported for 10 and 15% inclusion of rice DDGS without and with multi-enzymes are still poorer than reported in the present study which were as for 10% (1.69, 1.67) and 15% (1.71, 1.69). The values reported by Shirisha et al [23] on inclusion of 12 and 16% rice DDGS in broilers are also poorer than observed in the present study. The FCR reported by Singh et al [12] on 15% rice DDGS in broilers are consistent with the findings of present study. Wang et al [31] reported higher values for FCR when fed grain DDGS at higher levels in broilers. Thein et al [19] reported better FCR in broilers fed 20% rice DDGS compared to control. The comparatively lower FCR on higher level of rice DDGS in the present study may be attributed to lower availability of lysine in DDGS. The increased FCR on higher levels of rice DDGS suggests that the metabolizable energy value assigned to the DDGS might be an overestimate of its actual value or may be a result of the reduced bulk density of the diets with the higher levels of DDGS [32]; however, no mortality was recorded due to higher levels of rice DDGS in the present study.

### Nutrient metabolizability

The metabolizability of nutrients revealed non-significant differences for various nutrients (Table 4) except CP metabolizability, where it was higher (p<0.01) for CG than 15-RDDGS and 20-RDDGS groups; however, the CP metabolizability for protease supplemented 15 and 20 percent rice DDGS groups was comparable with control diet. The DM digestibility is consistent with Gupta [22] reported on 10% rice DDGS in broilers and Dinani et al [33] who reported similar trend with respect to inclusion of rice DDGS at 10%, 12.5% and 15% level; however, protein metabolizability reported by them is slightly lower than reported in the present study. The DM metabolizability and nitrogen retention of rice DDGS may be associated with level and type of crude fiber present and type of protein quality. The rice DDGS is high gross energy and protein feed but their availability to the body is limited. Better nutrient utilization may be associated with improved energy and protein digestibility by enzyme supplementation. Their supplementation hydrolyses fiber fraction, reduces the digesta viscosity and nutrient encapsulation, thereby increases nutrient utilization in the body [33]. The protease enzyme contributed to increase digestibility of starch and all other nutrients. Effects of protease on poultry diet do not appear to be completely limited to protein digestion, but also affect the digestibility of other nutrients. Increased digestion of corn starch with the use of protease is attributed to the disruption of protein matrix in starch granules.

The crude fiber components in the feed provide enormous influence on digestibility, the amount and composition. Cell content of fibrous feed almost everything can be digested, but the cell walls are composed of cellulose and hemicellulose are very difficult to digest because it contains a high lignin [34]. Some of the factors that affect the digestibility of crude fiber include fiber content in feed and the composition of the constituent crude fiber [35]. The observations on crude fiber metabolizability obtained in the present study falls under the same range.

The protease enzyme contributed to increase digestibility of starch and all other nutrients. Effects of protease on poultry diet do not appear to be completely limited to protein digestion, but also affect the digestibility of other nutrients. Increased digestion of corn starch with the use of protease is attributed to the disruption of protein matrix in starch granules. The protein hydrolysis catalyzed by the exogenous protease may be responsible for the improvement in apparent CP digestibility. The fiber degradation may be one of the mechanisms by which protease increases the digestion of nutrients in chickens [36]. Furthermore, it can be conceded that exogenous protease may exhibit its beneficial effects indirectly through maintenance requirement, secretion and recovery. Protease enzymes are added to broiler diets in an effort to increase protein utilization and digestion, which may enhance broiler performance. Studies have also revealed that broiler performance may not always be significantly improved by the addition of protease enzymes. The type and source of the protein in the broiler diet should also be taken into account. The performance of broilers may not be significantly affected by the addition of protease enzymes if the protein in the food is already of high quality and is easily digestible. However, the usage of protease enzymes may be more advantageous if the quality or digestibility of the protein in the diet is poor.

### Carcass traits

The carcass traits revealed non-significant differences for dressing percentage, overall giblet weight including liver, heart, gizzard and also for abdominal fat. The inclusion of rice DDGS up to 20 percent level does not have adverse effect on carcass traits in broilers also protease enzyme supplementation did not prove to be significantly beneficial in terms of carcass characteristics in broilers (Table 4). Our findings are in agreement with Dinani et al [37] who reported that inclusion of rice DDGS in diet did not show any significant variation in dressing percentage and eviscerated yield, abdominal fat and the internal organ weight *viz*. giblet (gizzard, heart, liver). It is also supported by the ICAR [38], which stated that adding rice DDGS up to 10% did not have a negative impact on the carcass attributes of broilers. Dinani et al [37] reported that enzyme supplementation showed no significant difference in carcass traits by feeding different levels of rice DDGS and rice gluten meal in combination. The findings are consistent with the present study not observing the effect of enzyme supplementation on carcass characteristics. They also revealed that organ weight in terms of giblet (gizzard, heart and liver) did not show any significant difference when fed different levels of rice DDGS as compared to control. Dang et al [24] reported non-significant differences in dressing percentage and organ weights when fed different levels of rice DDGS in chickens. In contrast, Wang et al [31] reported carcass yield and breast meat yield reduced when feeding maize DDGS levels greater than 15% of the diet.

### Gut health

The performance of the broiler is greatly influenced by the delicate and complex area of gut health. The caecal contents were examined for determination of *E. coli* and TVC count at 42^nd^ day were non-significant (Table 5). Dinani et al [39] did not show any significant difference in TVC due to different levels of rice DDGS. However, comparatively lower count for TVC and *E. coli* was observed due to 15 and 20 percent inclusion of rice DDGS in the present study. Also due to addition of enzymes the count further decreased than its counterpart both at 15 and 20 percent rice DDGS. The corn DDGS diet had a greater lactic acid concentration than the wheat DDGS diet and lactic acid is implicated to potentially inhibit the growth of pathogens [40]. On the other hand, the inclusion of RGM up to 20% level with or without protease enzyme supplementation in the diet revealed no significant effects on the intestinal microbiology of broiler chickens in the gut [41]. Due to recent changes in regulations regarding the use of antibiotics as feed additives, it appears that the association between food and intestinal microbiota is more significant in broiler chickens. It is important to research gut health, particularly the TVC and *E. coli* count in the digestive tract of broiler chickens, as poor gut health might impact digestion and nutrient absorption.

### Villi morphology

**Figure 1:**
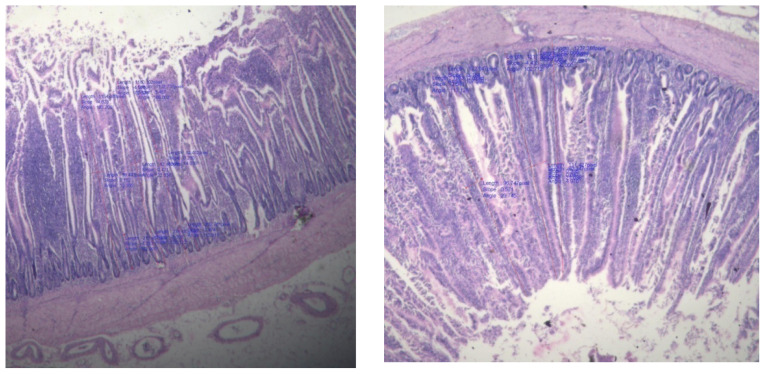
Villi height in duodenum.

**Figure 2:**
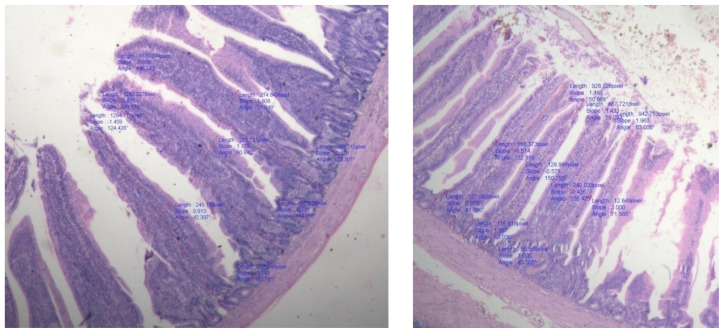
Villi height in jejunum.

**Figure 3:**
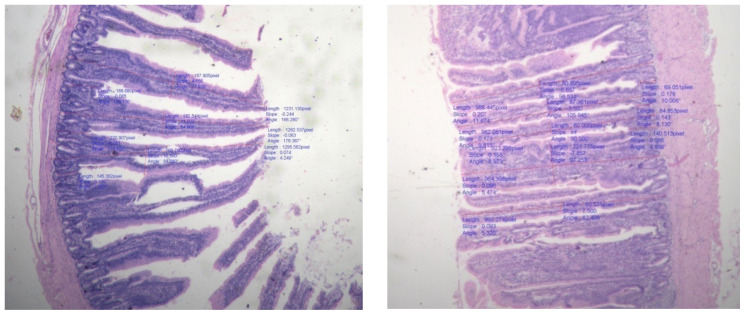
Villi height in ileum.

The small intestine is a crucial location for nutrient absorption from food. Due to an increase in absorptive surface area, improved gut structural morphology increases the intestine’s capacity for digestion and absorption. Due to its function in nutritional absorption, the small intestine is an essential component of the digestive system. So, it is considered crucial for this intestinal system to develop healthily in order to improve the health and productivity of broiler chickens. Villi and crypts are two important components of the small intestine and their geometry provides an indicator of the absorptive capacity of the small intestine [42]. Turnover of the intestinal epithelium reflects a dynamic equilibrium between the production of enterocytes in the crypts and their subsequent desquamation from the villus. The VH-to-crypt depth ratio is an available criterion for evaluating intestinal health and function [43]. On the 42^nd^ day of the experiment, the intestinal villi morphology in broilers from experimental groups was examined to determine the condition of the gut. The observations made are presented in Table 5 and Figures 1–3. The results revealed that no significant difference was observed in crypt depth (VD), villus width and VH among different dietary treatments and control group. Ranjan et al [16] reported no significant difference in villus length and crypt depth in duodenum, jejunum and ileum in ducks fed various levels of rice DDGS up to 75% replacement of soybean meal. The small intestine, especially the crypts and villi of the absorptive epithelium, plays significant roles in the final stages of nutrient digestion and assimilation [44]. No marked differences were observed in the average height of villi of the duodenum, jejunum and ileum by Ranjan et al [16] due to feeding of rice DDGS.

The DDGS addition maintains gut morphology and may play a role in processes of digestion and absorption of nutrients which is confirmed by the obtained data. In this respect, there are several possibilities for the gut to adapt or to react morphologically to change in diet composition or the intestinal microflora. Intestinal villi are often finger-like protrusions that expand the small intestine’s surface area to facilitate effective nutrient absorption. The villi shape, on the other hand, can be impacted and nutrient absorption decreased by the presence of specific feed ingredients or other variables. According to study, broilers fed a diet high in rice DDGS do not necessarily display modifications in villi morphology. It revealed that different levels of DDGS did not have significant effect on VH, width and crypt depth amongst the treatment groups. There was no negative impact on histological changes in intestine of interaction between DDGS and enzyme.

Information on effect of feeding diets containing various levels of rice DDGS with or without enzyme supplementation on histological changes is scanty. Available literature suggests that inclusion of protein source, corn DDGS in diet facilitate small intestine longitudinal growth in broilers, which may subsequently improve dietary nutrient absorption, whereas dietary treatments did not affect VH, width and crypt depth. In addition, broiler chicks with shallow intestinal crypts exhibited better growth performance. Rice based DDGS with and without enzyme has beneficial effect on gut health in terms of bacterial population and intestinal morphology [45].

### Economics

By taking into account the pricing of various inputs according to the market rates in effect at the time of the experiment, the economics of broiler production for all treatment groups were calculated. The prices of day-old chicks, feeds, medications, vaccinations, and other overhead costs were considered to complete the economics. The pre-starter, starter, and finisher feed rates for broilers were used to compute the total feed cost per kg. On a live body weight basis, the broilers were sold @ rate of Rs. 75/kg live body weight. For all treatment groups, additional costs such as labour, electricity, and other expenses were also regarded as uniform input. The details of economics are presented in Table 6.

The production cost was lowest for group including 20 percent rice DDGS, due to lower feed cost with higher inclusion of rice DDGS, followed by 20 percent rice DDGS supplemented with a protease enzyme. The production cost was highest for control group. The production cost for 15 percent rice DDGS group supplemented with enzyme was lower than that of without of enzyme. The net profit Rs/kg live body weight gain was highest for 15-RDDGSE group.

As the levels of rice DDGS were increased in broiler diet, the lesser feed costs per kg of feed were observed. The similar finding was observed by Choi et al [46] who observed that the use of DDGS in broiler diets up to 15% could decrease the feed cost by replacing part of corn and soybean meal, without any negative effect on growth performance and meat qualities. In the present study broilers fed diet containing the higher rice DDGS exhibited lowest feed cost. These findings are supported by researchers [15,37] who reported that feed cost per kg of live weight was highest in control without rice DDGS while the lowest feed cost was observed in the birds fed the diet containing higher levels of rice DDGS.

## CONCLUSION

The rice DDGS can be incorporated in broiler diet up to 20 percent level without any adverse effect on broiler performance maintaining gut health and optimized nutrient metabolizability. Supplementation of protease enzyme on inclusion of 15 and 20 percent DDGS in broiler diet proved to be beneficial in broilers. Broilers fed 15 percent rice DDGS supplemented with protease proved to be more economical.

## Figures and Tables

**Table 1:** Composition of different diets

Feed ingredients	CG			15% RDDGS			20% RDDGS		

	PS	ST	FS	PS	ST	FS	PS	ST	FS
Maize	57.50	59.00	61.00	58.25	60.10	62.40	58.50	60.00	62.50
Soybean meal	36.40	33.45	30.00	21.00	18.00	14.40	15.7	13.40	9.50
Rice DDGS	-	-	-	15.00	15.00	15.00	20.00	20.00	20.00
Vegetable oil	1.75	3.20	4.50	1.00	2.20	3.50	0.80	1.70	3.20
MCP	1.50	1.40	1.50	1.50	1.50	1.50	1.50	1.50	1.50
LSP	1.40	1.50	1.65	1.50	1.50	1.50	1.55	1.50	1.50
Salt	0.30	0.30	0.30	0.30	0.30	0.30	0.30	0.30	0.30
TMP	0.05	0.05	0.05	0.05	0.05	0.05	0.05	0.05	0.05
VP	0.05	0.05	0.05	0.05	0.05	0.05	0.05	0.05	0.05
DL-methionine	0.30	0.35	0.30	0.35	0.35	0.35	0.35	0.40	0.35
L-lysine	0.30	0.25	0.25	0.50	0.50	0.50	0.70	0.65	0.60
L-threonine	0.15	0.15	0.10	0.20	0.15	0.15	0.20	0.15	0.15
Choline chloride	0.10	0.10	0.10	0.10	0.10	0.10	0.10	0.10	0.10
Toxin binder	0.15	0.15	0.15	0.15	0.15	0.15	0.15	0.15	0.15
Coccidiostat	0.05	0.05	0.05	0.05	0.05	0.05	0.05	0.05	0.05

CG, control group; RDDGS, rice-based distillers’ dried grains with solubles; PS, pre-starter; ST, starter; FS, finisher; DDGS, distiller's dried grains with solubles; MCP, mono-calcium phosphate, LSP, limestone powder, TMP, trace mineral mixture (Fe 54 mg; I 1.2 mg; Cu 12 mg; Mn 90 mg; Zn 66 mg and Se 0.18 mg); VP, vitamin premix (Vitamin A 10,000 IU; vitamin D3 4,800 IU; vitamin E 45 mg; vitamin B_1_ 3 mg; vitamin B_2_ 9 mg; vitamin B_6_ 4.5 mg; vitamin B_12_ 40 μg; calcium panthotenate 16.5 mg; nicotinic acid 51 mg; folic acid 1.8 mg; biotin 0.15 mg).

**Table 2:** Proximate composition of different diets

Nutrients %	CG			15% RDDGS			20% RDDGS		

	PS	ST	FS	PS	ST	FS	PS	ST	FS
DM	91.00	90.86	91.12	91.00	90.52	90.84	90.98	90.64	91.06
CP	22.84	21.73	20.05	22.96	21.87	19.96	22.93	21.87	20.07
EE	3.32	3.96	5.44	3.82	4.12	5.78	4.06	4.24	5.96
CF	3.86	3.74	3.52	3.98	3.86	3.74	3.86	3.92	3.76
NFE	64.92	65.79	66.17	64.12	65.19	65.56	63.37	64.95	65.12
Total Ash	5.06	4.78	4.82	5.12	4.96	4.96	5.78	5.02	5.09
Calcium	1.12	1.16	1.16	1.09	1.17	1.15	1.19	1.12	1.21
Phosphorus	0.86	0.78	0.86	0.89	0.77	0.86	0.84	0.87	0.91
Methionine^1)^	0.65	0.61	0.56	0.69	0.71	0.71	0.68	0.72	0.66
Lysine^1)^	1.45	1.37	1.29	1.36	1.27	1.16	1.49	1.34	1.26
ME (Kcal/kg)^1)^	2,965	3,067	3,163	3,012	3,119	3,216	3,026	3,119	3,219

1) Calculated. The CP content of SBM–45.12%.

CG, control group; RDDGS, rice-based distillers’ dried grains with solubles; PS, pre-starter; ST, starter; FS, finisher; DM, dry matter; CP, crude protein; EE, ether extract; CF, crude fiber; NFE, nitrogen free extract, ME, metabolizable energy.

**Table 3:** Growth performance of broilers of rice DDGS

Growth phases	Groups					SEM	p-value

	CG	15-RDDGS	15-RDDGSE	20-RDDGS	20-RDDGSE		
Body weight gain (g)							
0–7 d	119^a^	106^bc^	110^b^	100^c^	106^bc^	1.64	0.001
8–14 d	793^a^	730^c^	766^ab^	690^c^	741^bc^	8.42	0.001
15–22 d	2,203^a^	2,093^bc^	2,071^b^	1,943^d^	1,942^cd^	28.67	0.001
Total	3,115^a^	2,930^bc^	2,947^b^	2,734^d^	2,789^cd^	37.38	0.001
Feed consumption (g)							
0–7 d	133	140	139	134	138	1.25	0.323
8–14 d	1,018^a^	970^abc^	1,001^ab^	941^d^	962^bc^	6.23	0.009
15–22 d	3,566^a^	3,485^b^	3,392^b^	3,287^c^	3,418^b^	26.60	0.001
Total	4,718^a^	4,594^ab^	4,532^ab^	4,394^c^	4,518^b^	32.35	0.002
Feed conversion ratio (FCR, feed to weight gain)							
0–7 d	1.11^b^	1.31^a^	1.25^a^	1.34^a^	1.30^a^	0.10	0.001
8–14 d	1.28^c^	1.33^b^	1.31^b^	1.37^a^	1.35^a^	0.09	0.009
15–22 d	1.61^c^	1.67^b^	1.64^b^	1.68^ab^	1.76^a^	0.08	0.005
Total	1.51^c^	1.57^b^	1.54^b^	1.58^b^	1.62^a^	0.07	0.009

a–d within the respective row differs significantly.

a,b means within variable with no common superscript differ significantly (p<0.05).

DDGS, distiller’s dried grains with solubles; CG, control group; RDDGS, rice-based distillers’ dried grains with solubles; RDDGSE, rice distillers dried grains with solubles supplemented with enzyme; SEM, standard error of the mean.

**Table 4:** Nutrient metabolizability and carcass traits in broilers on rice DDGS

Parameters	Groups					SEM	p-value

	CG	15-RDDGS	15-RDDGSE	20-RDDGS	20-RDDGSE		
Nutrient metabolizability %							
Dry matter	73	72	73	71	72	0.66	0.612
Ether extract	73	72	73	72	71	3.59	0.325
Crude protein	68^b^	58^a^	64^b^	56^a^	65^b^	2.88	0.005
Crude fiber	31	29	29	28	29	4.53	0.865
NFE	82	81	82	81	81	0.55	0.287
Carcass characteristics %							
Dressing%	71	70	70	69	69	0.62	0.139
Heart%	0.59	0.56	0.58	0.56	0.59	0.05	0.797
Liver%	1.80	1.87	2.01	1.99	1.87	0.10	0.779
Gizzard%	1.81	1.80	1.76	2.03	1.93	0.13	0.617
Giblet%	4.20	4.23	4.35	4.58	4.39	0.17	0.629
Abdominal fat	1.73	2.11	2.15	2.42	1.88	0.21	0.273

a,b within the respective row differes significantly.

DDGS, distiller’s dried grains with solubles; CG, control group; RDDGS, rice-based distillers’ dried grains with solubles; RDDGSE, rice distillers dried grains with solubles supplemented with enzyme; SEM, standard error of the mean; NFE, nitrogen free extract.

**Table 5:** Gut health of broilers on rice DDGS

Parameters	Groups					SEM	p-value

	CG	15-RDDGS	15-RDDGSE	20-RDDGS	20-RDDGSE		
*Escherichia coli* and TVC count							
*E. coli* 10^9^ cfu/g	6.50	5.50	5.25	5.50	5.25	1.41	0.208
TVC 10^9^ cfu/g	7.25	6.75	6.25	6.75	6.50	1.52	0.400
Villi morphometry duodenum (um)							
Villi height	1,606	1,346	1,829	1,685	1,587	83.27	0.349
Villi width	153	131	174	159	159	25.80	0.862
Crypt depth	295	205	177	258	201	39.86	0.322
Villi morphometry jejunum (um)							
Villi height	1,304	1,480	1,166	1,312	1,570	124.1	0.273
Villi width	186	201	154	187	180	29.49	0.892
Crypt depth	440	226	188	184	177	69.00	0.279
Villi morphometry ileum (um)							
Villi height	1,358	1,277	1,048	942	1,147	133.5	0.184
Villi width	196	175	190	146	196	21.55	0.585
Crypt depth	171	204	195	121	109	27.24	0.275

DDGS, distiller’s dried grains with solubles; CG, control group; RDDGS, rice-based distillers’ dried grains with solubles; RDDGSE, rice distillers dried grains with solubles supplemented with enzyme; SEM, standard error of the mean; cfu, colony forming unit; TVC, total viable count.

**Table 6:** Economics of broiler production on rice DDGS

Parameters	Groups				

	CG	15-RDDGS	15-RDDGSE	20-RDDGS	20-RDDGSE
Cost of chick (Rs)	36.00	36.00	36.00	36.00	36.00
Cost of rice DDGS (Rs/kg)	-	30.00	30.00	30.00	30.00
Protease supplementation (Rs/kg)	-	-	0.16	-	0.16
Cost of pre-starter feed (Rs/kg)	39.19	35.31	35.47	34.25	34.41
Cost of starter feed (Rs/kg)	38.74	35.17	35.33	33.94	34.10
Cost of finisher feed (Rs/kg)	38.44	35.29	35.45	34.10	34.26
Pre-starter feed consumed (g/bird)	133	140	139	134	138
Starter feed consumed (g/bird)	1,018	970	1001	931	962
Finisher feed consumed (g/bird)	3,566	3,485	3,392	3,257	3,418
Total feed consumed (g/bird)	4,718	4,595	4,532	4,322	4,518
Cost of total feed consumed (Rs.)	181.74	162.03	160.55	147.24	154.64
Miscellaneous (Rs)/bird	2.00	2.00	2.00	2.00	2.00
Production cost (Rs/bird)	219.74	200.03	198.55	185.24	192.64
Total body weight gain (g)	3,115	2,930	2,947	2,734	2,789
Selling rate (Rs./kg live BW)	75.00	75.00	75.00	75.00	75.00
Net profit (Rs./bird)	13.91	19.71	22.48	19.80	16.51
Net profit (Rs./kg live BW)	4.46	6.72	7.62	7.24	5.92

DDGS, distiller’s dried grains with solubles; CG, control group; RDDGS, rice-based distillers’ dried grains with solubles; RDDGSE, rice distillers dried grains with solubles supplemented with enzyme.
